# Reviewing the integration of patient data: how systems are evolving in practice to meet patient needs

**DOI:** 10.1186/1472-6947-7-14

**Published:** 2007-06-12

**Authors:** Ricardo J Cruz-Correia, Pedro M Vieira-Marques, Ana M Ferreira, Filipa C Almeida, Jeremy C Wyatt, Altamiro M Costa-Pereira

**Affiliations:** 1 Department of Biostatistics and Medical Informatics, Faculty of Medicine of University of Porto, Al. Prof. Hernâni Monteiro, 4200-319 Porto, Portugal; 2 Centre for Research in Health Technologies and Information Systems – CINTESIS (Centro de Investigação em Tecnologias e Sistemas de Informação em Saúde), Faculty of Medicine of University of Porto, Al. Prof. Hernâni Monteiro, 4200-319 Porto, Portugal; 3 Health Informatics Centre, University of Dundee, Dundee, Scotland, UK

## Abstract

**Background:**

The integration of Information Systems (IS) is essential to support shared care and to provide consistent care to individuals – patient-centred care. This paper identifies, appraises and summarises studies examining different approaches to integrate patient data from heterogeneous IS.

**Methods:**

The literature was systematically reviewed between 1995–2005 to identify articles mentioning patient records, computers and data integration or sharing.

**Results:**

Of 3124 articles, 84 were included describing 56 distinct projects. Most of the projects were on a regional scale. Integration was most commonly accomplished by messaging with pre-defined templates and middleware solutions. HL7 was the most widely used messaging standard. Direct database access and web services were the most common communication methods. The user interface for most systems was a Web browser. Regarding the type of medical data shared, 77% of projects integrated diagnosis and problems, 67% medical images and 65% lab results. More recently significantly more IS are extending to primary care and integrating referral letters.

**Conclusion:**

It is clear that Information Systems are evolving to meet people's needs by implementing regional networks, allowing patient access and integration of ever more items of patient data. Many distinct technological solutions coexist to integrate patient data, using differing standards and data architectures which may difficult further interoperability.

## Background

This review appraises studies examining the different approaches to integrating patient data from heterogeneous IS. Special attention is given to the type of integration engine and the type of integrated data. Articles published in the English literature between 1995 and 2005 with abstracts available were reviewed. We aimed to specifically review the integration of patient data, and how systems are evolving in practice to meet patient, professional and organisational needs.

A patient record is a set of documents containing clinical and administrative information regarding one particular patient, supporting communication and decision making in daily practice, and having different users and purposes [1]. Clinical care increasingly requires healthcare professionals to access patient record information that may be distributed across multiple sites, held in a variety of paper and electronic formats, and represented as mixtures of narrative, structured, coded and multimedia entries [2]. In hospitals, information technologies tend to combine different modules or subsystems, resulting in a best-of-breed approach [3]. Integration of healthcare Information Systems (IS) is essential to support shared care in hospitals, to provide proper care to mobile individuals and to make regional healthcare systems more efficient. However, to integrate clinical IS in a way that will improve communication and data use for healthcare delivery, research and management, many different issues must be addressed [4-6]. Consistently combining data from heterogeneous sources takes a great deal of effort because the individual feeder systems usually differ in several aspects, such as functionality, presentation, terminology, data representation and semantics [3]. It is still a challenge to make electronic health records interoperable because good solutions to the preservation of clinical meaning across heterogeneous systems remain to be explored [2]. Over the years different solutions to these problems have been proposed and some applied. Many of these solutions coexist in today's healthcare settings and are influenced by technology innovation and changes in healthcare delivery. Some of these solutions use differing standards and data architectures that may prove to be the greatest obstacle to semantic operability [7].

## Methods

### Eligible studies

Only studies describing or evaluating IS implementation for integrating patient data from heterogeneous IS were selected.

### Review team

The review team was composed of three Computer Scientists, namely Ana Margarida Ferreira, Pedro Vieira Marques, and Ricardo Cruz Correia, one medical doctor Filipa Canário Almeida advised by health informaticians experienced in systematic reviewing, Jeremy Crispin Wyatt and Altamiro Costa Pereira.

### Search methods

Studies were searched between September and October 2005 in the bibliographic databases. Since there is no specific standardised MeSH term, we developed a search string that includes the concepts of patient record, computers and data integration or sharing. Only articles with an abstract in English were considered. Given the significant evolution in ICT in the last decade, only studies published after 1994 (the last ten years) were included.

Three distinct bibliographic databases were searched: Medline (via Pubmed), ISI (ISI Web of Knowledge) and IEEE (IEEE Xplore). The query search string used in each database was ((medical or clinical or patient) and record*) and (comput* or digital or electronic*) and (integrat* or link* or sharing or share or shared).

This search method found 2443 articles in Pubmed, 961 in ISI and 414 in IEEE Xplore, a total of 3818 articles. After eliminating duplicate articles 3124 were selected.

### Selection of studies for the review

All four reviewers from the review team were involved in study selection. Six combinations of reviewer pairs were defined, due to the large number of articles found. The first selection was based on the study title. Each pair of reviewers read 512 titles. The study was considered eligible when at least one of the reviewers considered that the title mentioned one of three key concepts:

- Patient Records (e.g.: patient record, EPR, EHR, EMR, clinical documents – CDA, administrative database)

- Integration (e.g.: IS integration, record linkage, information sharing)

- Distributed environment (e.g.: e-Health, distributed healthcare, shared healthcare)

A total of 923 of 3124 articles were selected in this first selection on title alone.

The second phase of the study selection was based on abstracts. Again, six combinations of reviewer pairs were defined. Each pair of reviewers read 154 abstracts. The inclusion criterion in this phase was that articles should fulfil all three of the following conditions:

- Describe or assess IS implementations

- Integrate patient data from various IS

- Describe the technology used to integrate

**Figure 1:**
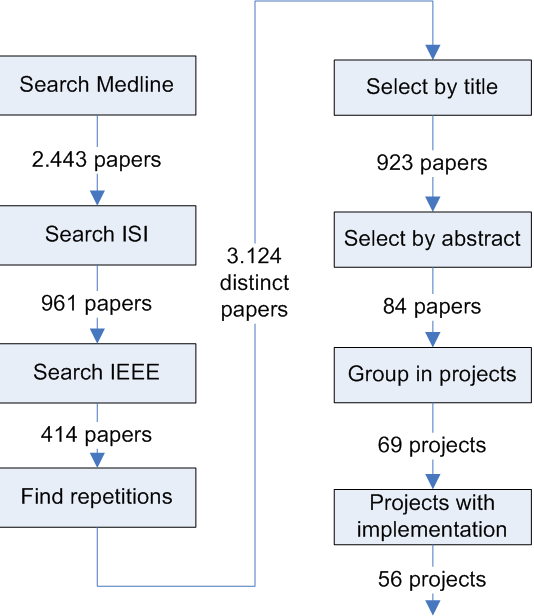
Diagram showing the methods used for study selection.

To maximize specificity, only selection by both reviewers was considered adequate. In cases of disagreement a third reviewer was called to decide. A total of 84 out of 923 articles were selected to be read entirely. These 84 articles were grouped into 69 distinct integration projects to avoid the distortion created by multiple papers describing the same project. All statistical analysis is based on projects and not on articles. Some of articles (n = 13) were descriptions of project plans or architecture models that were not already implemented on a real scenario nor even as a prototype. These projects were also excluded, leaving only 56 projects. Figure 1 is a flowchart illustrating the different stages of paper selection.

### Underlying model and definition of variables

**Figure 2:**
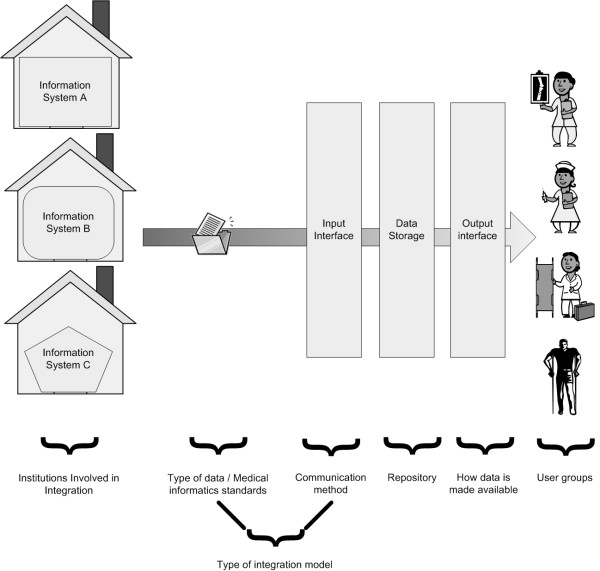
Framework for generic integration of heterogeneous Information Systems showing the stages and variables considered in the review.

Figure 2 illustrates the stages of a generic integration of heterogeneous IS. The variables examined in this review are related to these stages and intend to describe the context where the integration takes place (country, date, area covered, institutions involved, type of final users), the type of data integrated and the technology used (standards, communication methods, integration model, repositories of data, client applications).

The variables are:

- Country where the system is implemented;

- Date of article publication;

- Area covered by each project (country, region, hospital, department);

- Institutions involved as sources for patient data integration, i.e., institutions that own feeder systems to integration (departments, hospitals, primary care, private clinics, private labs, patient health portals) – multiple values are accepted;

- What type of medical data is integrated (lab orders, lab results, prescription orders, diagnosis or problems, procedures, admission letters, discharge letter, transfers letters, referral letters, medical images, biosignals) – multiple values are accepted;

- Medical informatics standards used (e.g.: HL7 – Health Level 7, CDA – Clinical Document Architecture, GEHR – Good European Health Record, SCIPHOX – Standardized Communication of Information Systems in Physician Offices and Hospitals using XML, DICOM -Digital Imaging and Communications in Medicine, MML – Medical Markup Language) – multiple values are accepted;

- Communication method (DICOM, DDE – Dynamic Data Exchange, e-mail, computer agents, Web services, Direct database access, CGI – Common Gateway Interface, CORBA – Common Object Request Broker Architecture, DHE – Distributed Healthcare Environment) – multiple values are accepted;

- Type of integration model for semantic interoperability (direct communication ie. when the systems create different interfaces to connect to each other; middleware ie. when an application programming interface is made available to talk with the central repository; semantic ie. when all possible data has a predefined message template, both semantic and syntax is known; generic ie. when the document structure accepts a certain degree of evolution without re-defining the whole template) – adapted from Bernstein et al. [8] – only one type of model is accepted;

- Type of data repository (File System, Database, PACS – Picture Archiving and Communication System, LDAP – Lightweight Directory Access Protocol, Virtual repository system) – multiple values are accepted;

- How data are made available to users (client application or web browser) – multiple values are accepted;

- How data are made available to other IS (Web services, CORBA or others) – multiple values are accepted;

- User groups (health professionals – medical users, nurses and other clinicians, clerical staff and patients) – multiple values are accepted;

### Time intervals considered

To analyse time trends, we divided the total period up into three shorter periods because of the small overall number of projects identified. The first period includes projects with their last publication in 1994–1999, the second period with their last publication in 2000–2002 and the third period with their last publication in 2003–2005.

### Statistical analysis

The statistical analysis was performed with SPSS^® ^version 14. *P *values in Table 1 were calculated using Pearson and linear-by-linear association chi-square tests with significance level of 0.05.

**Table 1:** Frequencies (and percentages) for each variable analysed among the 56 data integration projects reviewed

**Variable**	**n**	**(%)**	**Until 99 n = 7 **	**2000–2 n = 20 **	**2003–5 n = 29**	***p***^§^	**Projects numbers**
**Area covered by data integration**^†^							
Region or country	**35**	**(63)**	2 (29)	11 (55)	22 (76)		P 1, 2, 3, 4, 8, 10, 13, 14, 15, 16, 17, 19, 20, 21, 22, 27, 29, 31, 34, 35, 36, 38, 39, 40, 41, 42, 43, 44, 45, 47, 48, 50, 51, 52, 55
Hospital	**16**	**(29)**	4 (57)	7 (35)	5 (17)	.037	P 5, 6, 9, 11, 12, 18, 23, 24, 26, 32, 33, 37, 46, 53, 54, 56
Department	**5**	**(9)**	1 (14)	2 (10)	2 (7)		P 7, 25, 28, 30, 49
*Missing*	*0*	*(0)*	*0(0)*	*0(0)*	*0(0)*		

**Sources for patient data integration**^‡^							
Hospitals	**33**	**(69)**	4 (67)	13 (68)	16 (70)	*	P 2, 4, 5, 8, 10, 12, 13, 14, 15, 16, 20, 22, 23, 24, 27, 29, 31, 32, 34, 36, 37, 40, 41, 42, 43, 44, 47, 48, 50, 51, 53, 55, 56
Hospital departments	**19**	**(40)**	2 (33)	10 (53)	7 (30)	*	P 7, 9, 12, 14, 15, 21, 25, 26, 27, 28, 30, 31, 33, 37, 39, 46, 49, 53, 54
Primary care	**16**	**(33)**	0 (0)	5 (26)	11 (48)	.020	P 8, 12, 15, 16, 17, 22, 27, 29, 31, 34, 35, 38, 40, 41, 44, 48
Health portal	**4**	**(8)**	0 (0)	0 (0)	4 (17)	.054	P 13, 20, 34, 49
Private clinics	**1**	**(2)**	0 (0)	0 (0)	1 (4)	*	P 13
Private labs	**1**	**(2)**	0 (0)	0 (0)	1 (4)	*	P 13
*Missing*	*8*	*(14)*	*1(14)*	*1(5)*	*6(21)*		

**User groups**^‡^							
Health professionals	**42**	**(100)**	5 (100)	18 (100)	19 (100)		P 4, 8, 9, 10, 13, 14, 15, 17, 18, 20, 21, 22, 23, 24, 25, 26, 29, 30, 31, 32, 33, 34, 35, 36, 37, 38, 39, 40, 41, 42, 43, 44, 46, 47, 48, 49, 50, 51, 53, 54, 55, 56
Medical users	**20**	**(48)**	1 (20)	8 (44)	11 (58)	*	P 9, 10, 17, 18, 20, 25, 29, 35, 36, 37, 38, 40, 41, 43, 44, 48, 49, 54, 55, 56
Nurses	**4**	**(10)**	1 (20)	2 (11)	1 (5)	*	P 38, 42, 55, 56
Other health prof.	**5**	**(12)**	0 (0)	4 (22)	1 (5)	*	P 10, 18, 31, 42, 44
Patients	**2**	**(5)**	0 (0)	0 (0)	2 (11)	*	P 13, 38
Clerical	**1**	**(2)**	0 (0)	0 (0)	1 (5)	*	P 22
*Missing*	*14*	*(25)*	*2(29)*	*2(10)*	*10(34)*		

**Type of data integrated**^‡^							
Diagnosis and Problems	**40**	**(77)**	6 (86)	13 (76)	21 (75)	*	P 1, 2, 3, 5, 8, 9, 10, 11, 12, 14, 15, 16, 20, 21, 22, 23, 24, 25, 26, 27, 29, 30, 31, 32, 34, 36, 37, 38, 40, 41, 42, 43, 46, 47, 49, 50, 51, 53, 54, 55
Images	**35**	**(67)**	4 (57)	14 (82)	17 (61)	*	P 3, 6, 8, 9, 10, 11, 12, 13, 14, 15, 16, 20, 21, 22, 25, 26, 27, 28, 30, 31, 32, 33, 36, 37, 39, 42, 43, 44, 46, 47, 48, 49, 51, 54, 56
Lab results	**34**	**(65)**	6 (86)	11 (65)	17 (61)	*	P 1, 2, 8, 9, 10, 11, 12, 13, 14, 15, 16, 18, 21, 22, 23, 24, 26, 27, 28, 29, 32, 34, 36, 37, 39, 40, 42, 43, 45, 47, 51, 54, 55, 56
Discharge	**33**	**(63)**	2 (29)	12 (71)	19 (68)	*	P 1, 8, 9, 10, 11, 12, 13, 14, 16, 17, 18, 20, 21, 22, 23, 24, 25, 27, 29, 34, 37, 39, 40, 41, 42, 44, 47, 50, 51, 52, 53, 54, 56
Procedures	**31**	**(60)**	4 (57)	9 (53)	18 (64)	*	P 1, 2, 3, 8, 9, 10, 11, 12, 15, 16, 21, 22, 25, 26, 27, 29, 32, 34, 36, 40, 42, 43, 44, 46, 47, 49, 50, 51, 53, 54, 55
Prescription	**28**	**(54)**	4 (57)	8 (47)	16 (57)	*	P 1, 2, 8, 10, 11, 12, 13, 17, 22, 23, 24, 25, 27, 29, 32, 38, 40, 42, 43, 44, 45, 47, 48, 51, 53, 54, 55, 56
Admission	**25**	**(48)**	3 (43)	7 (41)	15 (54)	*	P 1, 2, 3, 10, 11, 12, 13, 14, 18, 20, 22, 23, 24, 27, 29, 37, 39, 41, 42, 45, 47, 50, 52, 53, 54
Transfers	**16**	**(31)**	2 (29)	6 (35)	8 (29)	*	P 1, 10, 11, 12, 18, 20, 22, 23, 24, 29, 37, 42, 47, 50, 53, 54
Referrals	**10**	**(19)**	0 (0)	3 (18)	7 (25)	*	P 1, 15, 22, 27, 34, 37, 38, 41, 47, 52
Lab orders	**9**	**(17)**	0 (0)	3 (18)	6 (21)	*	P 1, 10, 12, 13, 18, 22, 27, 42, 51
Bio-signal	**9**	**(17)**	1 (14)	6 (35)	2 (7)	*	P 3, 22, 27, 42, 44, 51, 53, 54, 56
*Missing*	*4*	*(7)*	*0(0)*	*3(43)*	*1(3)*		

**Type of model**^†,ℓ^							
Semantic – all data structured	**21**	**(44)**	1 (20)	8 (47)	12 (46)		P 5, 9, 13, 15, 16, 20, 21, 22, 25, 29, 30, 33, 34, 37, 38, 40, 50, 52, 54, 55, 56
Middleware	**19**	**(40)**	3 (60)	8 (47)	8 (31)		P 4, 11, 14, 18, 19, 24, 26, 27, 31, 32, 39, 41, 43, 44, 46, 47, 48, 49, 51
Direct communication	**5**	**(10)**	1 (20)	1 (6)	1 (12)		P 2, 6, 7, 23, 45
Generic – structure and data dynamic	**3**	**(6)**	0 (0)	0 (0)	3 (12)		P 1, 17, 36
*Missing*	*7*	*(13)*	*2(29)*	*3(15)*	*3(10)*		

**Medical informatics standards**^‡^							
HL7 (includes CDA)	**23**	**(68)**	2 (67)	12 (80)	9 (56)	*	P 3, 4, 5, 9, 11, 14, 16, 21, 27, 29, 30, 34, 35, 37, 40, 41, 42, 44, 50, 52, 54, 55, 56
Just CDA	**5**	**(15)**	0 (0)	1 (7)	4 (25)	*	P 16, 21, 27, 34, 52
DICOM	**11**	**(32)**	2 (67)	6 (40)	3 (19)	.071	P 3, 9, 14, 25, 31, 32, 34, 35, 43, 44, 51
Other	**8**	**(24)**	2 (67)	3 (20)	3 (19)	*	P 3, 27, 32, 33, 35, 38, 47, 51
GEHR	**3**	**(9)**	1 (33)	0 (0)	2 (13)	*	P 3, 17, 36
SCIPHOX	**1**	**(3)**	0 (0)	0 (0)	1 (6)	*	P 34
MML	**1**	**(3)**	0 (0)	0 (0)	1 (6)	*	P 13
*Missing*	*24*	*(43)*	*4(57)*	*5(25)*	*15(52)*		

**Repository**^‡^							
Database	**34**	**(77)**	5 (83)	10 (63)	19 (86)	*	P 2, 4, 7, 9, 12, 13, 16, 18, 20, 22, 23, 24, 25, 26, 27, 29, 31, 32, 33, 38, 39, 40, 41, 43, 44, 45, 46, 47, 48, 49, 50, 54, 55, 56
Virtual	**11**	**(25)**	1 (17)	7 (44)	3 (14)	*	P 5, 6, 8, 14, 21, 28, 35, 37, 42, 49, 54
Files	**7**	**(16)**	1 (17)	2 (13)	4 (18)	*	P 9, 10, 20, 22, 26, 31, 38
PACS	**1**	**(2)**	0 (0)	0 (0)	1 (5)	*	P 25
LDAP	**1**	**(2)**	0 (0)	0 (0)	1 (5)	*	P 27
*Missing*	*13*	*(23)*	*1(14)*	*4(20)*	8(28)		

**Communication method**^‡^							
Database direct access	**9**	**(30)**	4 (80)	1 (13)	4 (24)	.063	P 2, 23, 26, 28, 32, 39, 41, 46, 54
Web services	**8**	**(27)**	0 (0)	1 (13)	7 (41)	.042	P 5, 6, 16, 20, 33, 38, 49, 52
CORBA	**4**	**(13)**	0 (0)	2 (25)	2 (12)	*	P 14, 27, 33, 47
E-Mail	**3**	**(10)**	0 (0)	1 (13)	2 (12)	*	P 15, 20, 34
DICOM	**3**	**(10)**	0 (0)	1 (13)	2 (12)	*	P 25, 31, 43
DDE	**3**	**(10)**	0 (0)	2 (25)	1 (6)	*	P 7, 14, 34
DHE	**3**	**(10)**	0 (0)	2 (25)	1 (6)	*	P 19, 47, 48
CGI	**3**	**(10)**	2 (40)	0 (0)	1 (6)	.082	P 32, 39, 55
Agents	**2**	**(7)**	1 (20)	0 (0)	1 (6)	*	P 11, 26
*Missing*	*28*	*(50)*	*2(29)*	*12(60)*	*14(48)*		

**How data is made available to users**^‡^							
Web browser	**34**	**(92)**	5 (83)	11 (100)	18 (90)	*	P 2, 3, 5, 6, 9, 10, 13, 15, 16, 17, 18, 19, 20, 23, 25, 26, 27, 29, 31, 32, 35, 36, 38, 39, 41, 42, 43, 44, 45, 47, 48, 49, 55, 56
Client application	**7**	**(19)**	1 (17)	3 (27)	3 (15)	*	P 9, 12, 13, 24, 33, 48, 51
*Missing*	*19*	*(34)*	*1(14)*	*9(45)*	*9(31)*		

**How data is made available to other Information Systems**^‡^							
Web services	**7**	**(88)**	0 (-)	2 (100)	5 (83)		P 6, 11, 16, 18, 27, 35, 49
CORBA	**1**	**(13)**	0 (-)	0 (0)	1 (17)	*	P 27
*Missing*	*48*	*(86)*	*7(100)*	*18(90)*	*23(79)*		

†: single variable with mutually exclusive response categories

‡: multiple variables with dichotomous response categories (yes or no)

*: not statistically significant

§: linear-by-linear association chi-square test used (except in variable type of model)

ℓ:models used for semantic interoperability: **direct communication **when the systems create different interfaces to connect; **middleware **when an API is used to talk with the central repository; **semantic **when all data has a predefined message template; **generic **when the document structure accepts evolution without re-defining the whole template. *P *value calculated using Pearson association chi-square test.

## Results

### Study selection

The agreement rate for the first phase was 83%, and for the second phase was 77%. The number of different IS implemented was 56. Table 2 lists all integrated IS considered in this review, their country, number of publications and period of publication. Countries with the most published projects were the USA (15), Germany (8), Greece (6), Denmark (4) and China (4). Most IS (73%) have just one publication. 52% of the IS had their last publication in the period 2003–5, and 36% during 2000–2.

**Table 2:** Integrated IS included in the review, country in which installed, number and date of publications.

**Project number**	**System name (or location)**	**Country**	**Number of publications**	**Publication dates**	**References**
P1	Aarhus County	Denmark	1	2005	[8]
P2	Allina	USA	1	2004	[13]
P3	Argonauta	Germany	1	1999	[14]
P4	CareHaven	China	1	2001	[15]
P5	CareWeb	USA	2	[1998, 2000]	[16, 17]
P6	Chili	Germany	1	2004	[18]
P7	Cleveland – USA	USA	1	2000	[19]
P8	Clicks	Israel	1	2003	[20]
P9	Clinical Desktop (former Spectrum)	USA	1	2002	[21]
P10	Clinical Management System	China	1	2005	[22]
P11	Daegu – Korea	Korea	1	2003	[23]
P12	DIOGENE	Switzerland	5	[1998, 2005]	[24–28]
P13	Dolphin	Japan	1	2005	[9]
P14	Epirus-net	Greece	1	2001	[29]
P15	Funen Health Care Network	Denmark	1	2000	[30]
P16	GDGHA – General Hospital of Athens	Greece	4	[2002, 2004]	[31–34]
P17	GP Software Integration Project	Australia	1	2003	[35]
P18	GTDS	Germany	1	2001	[36]
P19	H:S	Denmark	1	2005	[8]
P20	health@net	Austria	2	[2004, 2005]	[37, 38]
P21	Heilderberg	Germany	1	2001	[39]
P22	HELP 2	USA	1	2003	[40]
P23	Henri Mondor University Hospital	France	1	1999	[41]
P24	HIS/BUI	Israel	1	1998	[42]
P25	Hong Kong Polyt. Univ.	China	1	2005	[43]
P26	Image Engine	USA	1	1996	[44]
P27	HYGEIAnet	Greece	5	[2000, 2004]	[45–49]
P28	IHIS	Greece	1	1997	[50]
P29	INPC (new version of RMRS)	USA	2	[2001, 2004]	[51, 52]
P30	Magdburg	Germany	1	2002	[53]
P31	MedSec	Germany	1	2001	[54]
P32	MINDscape	USA	1	1998	[55]
P33	MUDR	Czech Republic	1	2004	[56]
P34	Munster	Germany	5	[2001, 2005]	[57–61]
P35	National Tech Univ. Athens	Greece	1	2002	[62]
P36	OACIS	Australia	1	2003	[35]
P37	OITL	USA	1	2001	[63]
P38	old@home	Sweden	1	[2004, 2005]	[10, 64]
P39	Oxford Clinical Intranet	UK	2	[1999, 2004]	[65, 66]
P40	PeaceHealth	USA	1	2004	[67]
P41	PHIN – Public Health Information Network	USA	1	2004	[68]
P42	RMRS – Regenstrief	USA	3	[1999, 2001]	[69–71]
P43	Shanghai Hospital	China	1	2005	[72]
P44	SPERIGEST	Italy	5	[1997, 2002]	[73–77]
P45	SUP	Denmark	1	2005	[8]
P46	Sydney	Australia	1	2005	[78]
P47	Synapses	Ireland	4	[1997, 2001]	[79–82]
P48	SynEx@UBSC	Italy	2	[1999, 2002]	[83, 84]
P49	TACWeb	Italy	1	2003	[85]
P50	Thessaloniki	Greece	1	2003	[86]
P51	Veterans Affairs	USA	2	[1999, 2000]	[87, 88]
P52	V-Net Med	Germany	1	2005	[89]
P53	Virgin del Rocio University Hospital – Seville	Spain	1	2002	[90]
P54	Web/VS	USA	1	2001	[91]
P55	WebCIS	USA	1	1999	[92]
P56	Web-EPR	Brazil	1	2001	[93]

### Trends

#### Area covered by integration

59% of the IS covered only a region, while 29% covered a hospital, 9% a department and 4% a whole country. There was a downward trend in publications related to projects that cover a hospital from 57% until 1999, 35% in 2000–02 and 17% in 2003–05. The number of projects covering a region or country has increased over the years, and currently represents 76% (p = 0.037).

#### Institutions involved in the integration

Most of the integrated information comes from hospital IS (69%), with departmental (40%) and primary care (33%) IS representing the next two most frequent institution types. Four projects (8%) integrated information from health portals; all were published in the most recent period considered (2003–05).

#### User groups

As expected, all information systems provided access to health professionals. Two recent projects claim giving data access to patients [9,10]. Medical doctors are more often referenced as users (48%) than nurses (10%).

#### Integrated data

77% of the projects integrated diagnosis and problems, 67% medical images, 65% lab results, 63% discharge notes and 60% procedures. There has been an increase in projects integrating referral letters (from 0% until 1999, to 18% in 2000–02 and to 25% in 2003–05).

#### Type of models

Regarding the type of integration model, although the number of projects found using a predefined message templates (semantic – all data structured) and middleware are very similar (44% and 40% respectively), it seems that there is a trend to use more predefined message templates (46% in 2003–05) and fewer middleware solutions (31% in 2003–05). This tendency is clearer, if the values of the projects using messaging (both "Semantic – all data structured" and "Generic – structure and data dynamic") are added, representing 54% in 2003–05. Direct communication to databases is very low (10%) and more flexible messaging is now appearing (12% in 2003–05).

#### Messaging standards

HL7 is the most frequently used messaging standard (68%). It seems that CDA is becoming the reference to use inside HL7 (25% in 2003–05). DICOM is becoming less used when compared to other standards, which is understandable as it is mainly for images. Nevertheless, DICOM is no more the only success example of standards use in medical communication protocols. Other standards have very low usage nowadays (19% in 2003–05).

#### Repository

Regarding the type of data storage, 77% of the projects stored data in databases, 25% used virtual repositories and 16% stored in files. There is no real change over the periods considered.

#### Communication method

Recently (since 2000) more different technologies have been used to establish communication (3 until 1999, 8 in 2000–02 and again 8 in 2003–05). Web services have increasing importance (p = 0.042), whilst Database direct access and Common Gateway Interface have decreasing importance.

#### How data are made available for users

92% of the Information Systems use a Web browser to deploy their applications, whilst only 19% give user access through client-server applications.

#### How data are made available for other IS

88% of the IS use Web services to communicate with other systems, whilst only 13% use CORBA. The absolute number of systems using Web Services has grown from zero until 1999, two in 2000–2 and 5 in 2003–05.

### Current status (results regarding 2003–05)

Currently there are more projects carrying out regional integration, especially between hospitals and primary care. Referral letters are mentioned in 7 of the 29 projects described in articles published in 2003–05. It is also clear that patients are also becoming active participants because they appear for the first time as a user group in more recent projects.

Regarding integration models, messaging between systems, both Semantic and Generic, is lately used more frequently (58%) than middleware (31%). Databases are still the most common method for data storage (86%). Communication between integrated systems uses many different technologies with Web services being used in 41% of the projects. The most common user interface by far is the Web browser (90%).

## Discussion

Our results show an increasing number of publications describing projects which integrate data from multiple Information Systems. This is in agreement with our initial assumption about the interest in improving the communication of health related data to support person-centred healthcare. As the number of heterogeneous health IS grows, their integration becomes a priority. Moreover, we may be witnessing an increasing interest in regional integration between heterogeneous healthcare information systems across different institutions, to help communication between the different stake holders (primary and secondary care doctors, nurses and patients). This is also supported by the increasing communication of referral letters.

It should be noticed the efforts being put into integration in countries like Germany, Greece and Denmark which are trying to implement nationwide healthcare integrated networks feed by heterogeneous information systems.

Messaging technologies (in particular HL7) are more used than middleware solutions (like DCOM or CORBA). Web based technologies (web-services and web-browsers) support most of the projects, indicating that these new technologies are quickly adopted in healthcare institutions. Nevertheless, it is obvious that many distinct technological solutions coexist to integrate patient data.

The concept of message passing appears to be radically different from the conventional concept of procedure calls or operation invocation, but the difference is more one of pedagogical emphasis than of semantics. Message passing emphasizes the remoteness of the object and the caller's lack of knowledge of the code body which will be executed. However, any procedure call can be viewed as an exchange of messages [11]. The main difference is both approaches is the reliance on open Internet standards like HTTP, XML, SOAP, WSDL, UDDI and WSFL by the Web services (messaging), in opposition to DCOM and CORBA solutions (middleware) that resulted many times in single-vendor implementation requirements.

One key omission from the literature reviewed is that most of the project publications failed to mention any type of error detection. We feel that is mandatory to verify the quality of integrated data, so that instead of propagating data errors, alerts regarding data quality can be triggered and correction processes can take place [12].

### Limitations

One of the main limitations of this review is lack of detail reported in most of the articles, and especially the non existence of any impact evaluation of the technologies they describe, despite the enormous cost of such systems and the evident change in working practices that they entail. The percentage of missing values for each time interval varied between 0 and nearly 50% depending on the type of variable analysed and interval of time considered.

Another limitation is only considering papers published in the last ten years may exclude early work on integration at the hospitals, although we feel it is justifiable given the significant evolution in ICT in the last decade.

Although we feel that grouping the papers into projects is essential to decrease the bias of multiple publications of the same project, on some of the papers it was difficult to determine if they were describing the same project or not.

## Conclusion

Currently people have more mobility, longer lives and health care is more shared than ever before. It is clear that Information Systems are evolving to meet people's needs by implementing regional networks, allowing patient access and integration of ever more items of patient data. We conclude that patient information is becoming more accessible as there are more integrated IS which are more likely to involve primary care and a wider range of patient data.

Web based technologies and messaging technologies are supporting most of the current integration projects, indicating that these new technologies are quickly adopted in healthcare institutions. Many distinct technological solutions coexist to integrate patient data, using differing standards and data architectures which may difficult further interoperability.

## Competing interests

The author(s) declare that they have no competing interests.

## Authors' contributions

RCC was responsible for the study design, organization, analyses and manuscript preparation. RCC, AMF, PVM and FCA were part of the articles review team. JCW and ACP advised the study design and supervised statistical analysis and the manuscript preparation. All authors read and approved the final manuscript.

## Pre-publication history

The pre-publication history for this paper can be accessed here:


